# Prediction and characterization of promoters and ribosomal binding sites of *Zymomonas mobilis* in system biology era

**DOI:** 10.1186/s13068-019-1399-6

**Published:** 2019-03-14

**Authors:** Yongfu Yang, Wei Shen, Ju Huang, Runxia Li, Yubei Xiao, Hui Wei, Yat-Chen Chou, Min Zhang, Michael E. Himmel, Shouwen Chen, Li Yi, Lixin Ma, Shihui Yang

**Affiliations:** 1 State Key Laboratory of Biocatalysis and Enzyme Engineering, Hubei Collaborative Innovation Center for Green Transformation of Bio-resources, Environmental Microbial Technology Center of Hubei Province, and School of Life Sciences, Hubei University, Wuhan, 430062 China; 2 Biosciences Center, National Renewable Energy Laboratory, Golden, CO 80401 USA; 3 National Bioenergy Center, National Renewable Energy Laboratory, Golden, CO 80401 USA

**Keywords:** *Zymomonas mobilis*, Systems biology, Promoter, Ribosomal binding site (RBS), Reporter genes, Dual reporter-gene system

## Abstract

**Background:**

*Zymomonas mobilis* is a model bacterial ethanologen with many systems biology studies reported. Besides lignocellulosic ethanol production, *Z. mobilis* has been developed as a platform for biochemical production through metabolic engineering. However, identification and rigorous understanding of the genetic origins of cellular function, especially those based in non-coding region of DNA, such as promoters and ribosomal binding sites (RBSs), are still in its infancy. This knowledge is crucial for the effective application of *Z. mobilis* to new industrial applications of biotechnology for fuels and chemicals production.

**Results:**

In this study, we explored the possibility to systematically predict the strength of promoters based on systems biology datasets. The promoter strength was clustered based on the expression values of downstream genes (or proteins) from systems biology studies including microarray, RNA-Seq and proteomics. Candidate promoters with different strengths were selected for further characterization, which include 19 strong, nine medium, and ten weak ones. A dual reporter-gene system was developed which included appropriate reporter genes. These are the *opmCherry* reporter gene driven by the constitutive P*lacUV5* promoter for calibration, and *EGFP* reporter gene driven by candidate promoters for quantification. This dual reporter-gene system was confirmed using the inducible promoter, P*tet*, which was used to determine the strength of these predicted promoters with different strengths. In addition, the dual reporter-gene system was applied to determine four synthetic RBSs with different translation initiation rates based on the prediction from bioinformatics server RBS calculator. Our results showed that the correlations between the prediction and experimental results for the promoter and RBS strength are relatively high, with *R*^2^ values more than 0.7 and 0.9, respectively.

**Conclusions:**

This study not only identified and characterized 38 promoters and four RBSs with different strengths for future metabolic engineering in *Z. mobilis*, but also established a flow cytometry-based dual reporter-gene system to characterize genetic elements including, but not limited to the promoters and RBSs studied in this work. This study also suggested the feasibility of predicting and selecting candidate genetic elements based on omics datasets and bioinformatics tools. Moreover, the dual reporter-gene system developed in this study can be utilized to characterize other genetic elements of *Z. mobilis*, which can also be applied to other microorganisms.

**Electronic supplementary material:**

The online version of this article (10.1186/s13068-019-1399-6) contains supplementary material, which is available to authorized users.

## Background

With the increasing consumption of fossil energy, significant efforts have focused on the development of sustainable alternative renewable energy needed to meet the energy demands for economic development and environmental protection. Correspondingly, biofuels produced through biochemical conversion from biomass-based feedstock by microorganisms have attracted significant attention. *Z. mobilis* is a natural ethanologenic bacterium with many desirable characteristics necessary to produce lignocellulosic biofuels and their intermediates through metabolic engineering, including ethanol and 2,3-butanediol [1–4].

To meet the needs of metabolic engineering and synthetic biology approaches, *Z. mobilis* genetic elements from coding regions (genes) and non-coding regions [e.g., promoters, ribosomal binding site (RBS), untranslated region (UTR), and terminators] have been broadly investigated [5]. Different from those in the coding region, genetic elements from the non-coding region can affect gene expression at the transcriptional or translational levels; as well as modulate their activity in response to environmental conditions [6–9]. Although some non-coding sequence elements of *Z. mobilis* were discovered [7, 10], there are no systematic and efficient approaches to identify and quantify elements that were already discovered. Many genetic elements remain undiscovered today.

With the development and deployment of technology, such as next-generation sequencing (NGS) and mass spectrometry, many systems biology studies were carried out with enormous omics data accumulated [11–13], including studies of *Z. mobilis* [13–27]. These systems biology datasets are vast, and thus contain information useful for deep mining and modeling [28, 29]. For example, genetic elements such as promoters can be sorted out by multiple omics data analysis [30]. However, promoters of *Z. mobilis* have not been systematically characterized, although the accurate information of genome and plasmid sequence; as well as genome annotation of *Z. mobilis* are available [14, 31, 32].

Traditionally, native promoters and RBSs are often discovered as a result of random genomic digestion, which is further facilitated by genome sequencing and annotation [6]. These genetic elements of different strengths are often required in metabolic engineering practices. For example, strong promoters were usually used to overexpress target genes to increase the titer of heterologous products [33]. Up to now, only a small number of strong promoters, such as P*gap*, P*pdc*, and P*eno*, have been investigated and used to construct metabolic pathways into wild-type strains suitable for efficient xylose utilization or enhanced inhibitor tolerance in *Z. mobilis* [34–43].

The application of strong promoters on metabolic engineering could cause metabolic burden on cellular growth, and thus reduce the titer, yield, or productivity. To address this issue, inducible promoters, such as P*tet*, were recently used to direct metabolic flux for balanced cellular growth and production [44]. However, the strengths of these promoters have not been investigated quantitatively, making it difficult to meet the needs of metabolic engineering and synthetic biology for genetic elements with different strengths [45]. Moreover, no RBS sequences have been specifically studied for *Z. mobilis*.

Some in vitro methods, such as electrophoresis mobility shift assay (EMSA) [46] and atomic force microscope (AFM) [47], have been developed to characterize promoter activity, but are not widely used due to their low accuracy in characterization of genetic elements quantitatively in vivo. The classical strategy to quantify the promoter activity in vivo is to measure the expression of downstream reporter genes driven by the candidate promoter [48]. Reporter genes, such as β-glucuronidase, β-galactosidase and fluorescent proteins, are commonly used for this purpose.

Fluorescent reporter proteins, which were established in 1996 [49] and developed later [50, 51], are preferred to be used with their unique advantages, which include being relatively small and nontoxic [52]. However, the expression of fluorescent reporter genes is affected by many internal and external factors, including mRNA degradation, translation, protein folding; as well as plasmid copy numbers [53], which then influence the promoter characterization in vivo [54]. These combined factors make it difficult to accurately measure the promoter activity in vivo, especially when using a single report gene in individual experiment without an intrinsic control [55, 56]. To reduce these influences, a dual reporter-gene system was developed and used for promoter characterization in different species, such as *E. coli*, *Clostridium* sp., and virus [55, 57, 58].

In this study, a dual reporter-gene system was developed which contains: (1) the *opmCherry* reporter gene driven by the constitutive *PlacUV5* promoter for calibration, and (2) the *EGFP* reporter gene for quantifying the candidate genetic elements located upstream of *EGFP*. A series of promoters with different strengths were predicted based on previous systems biology data and four synthetic RBSs with different translation initiation rates were predicted and synthesized. These promoters and RBSs were then experimentally characterized using the dual reporter-gene system to explore the relationship between the prediction and experimental data.

## Results

### Establishment of the dual reporter-gene system

**Fig. 1:**
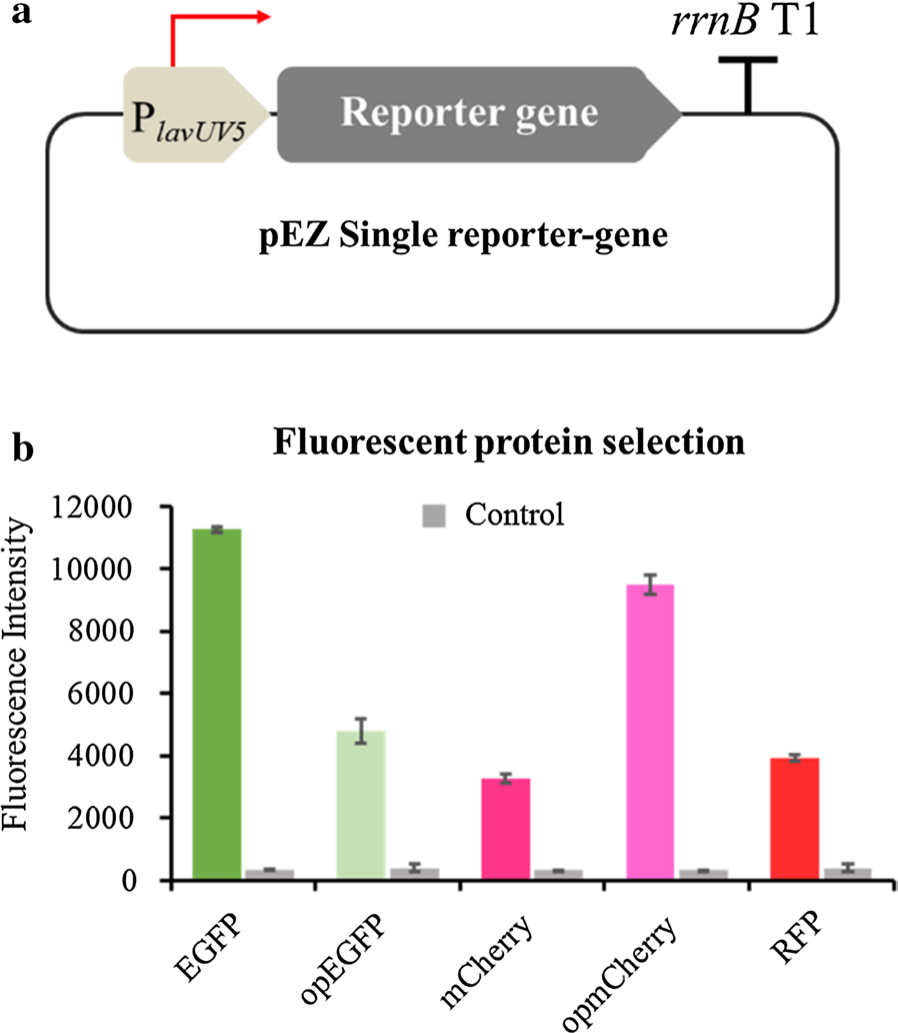
The schematic illustration of the reporter-gene system including a constitutive promoter Plac*UV5* to drive the reporter gene to be characterized and a terminator of *rrnB* gene (**a**), and the fluorescence intensity of selected fluorescent proteins of EGFP, opEGFP, mCherry, opmCherry and RFP driven by a strong promoter *PlacUV5* (**b**). The emission/excitation wavelength (nm) of fluorescence protein: EGFP and opEGFP (488/507), mCherry and opmCherry (587/610), as well as RFP (555/584)

To develop a reliable dual reporter-gene system, fluorescent protein pairs should meet some requirements. Firstly, the pair should have distinguishable excitation and emission spectrum to avoid interplay between channels. Secondly, similar mature time rates are preferred, since different mature time rates will affect the simultaneous measurement. Thirdly, bright fluorescence intensity is preferred to help detect the signal intensity of relatively weak promoters. Based on these criteria, seven single fluorescent proteins, including EGFP, mCherry, RFP and CFP; as well as codon-optimized EGFP (opEGFP), mCherry (opmCherry), and CFP (opCFP), were synthesized and characterized under the control of a constitutive promoter P*lacUV5* using the shuttle vector pEZ15Asp [44] (Fig. 1a). The emission and excitation wavelength of CFP were 433 nm and 475 nm, which was failed to detect by our detector and not included in this work. Flow cytometry results indicated that EGFP and opmCherry produced the brightest fluorescence signal among the seven fluorescent proteins tested (Fig. 1b).

**Fig. 2:**
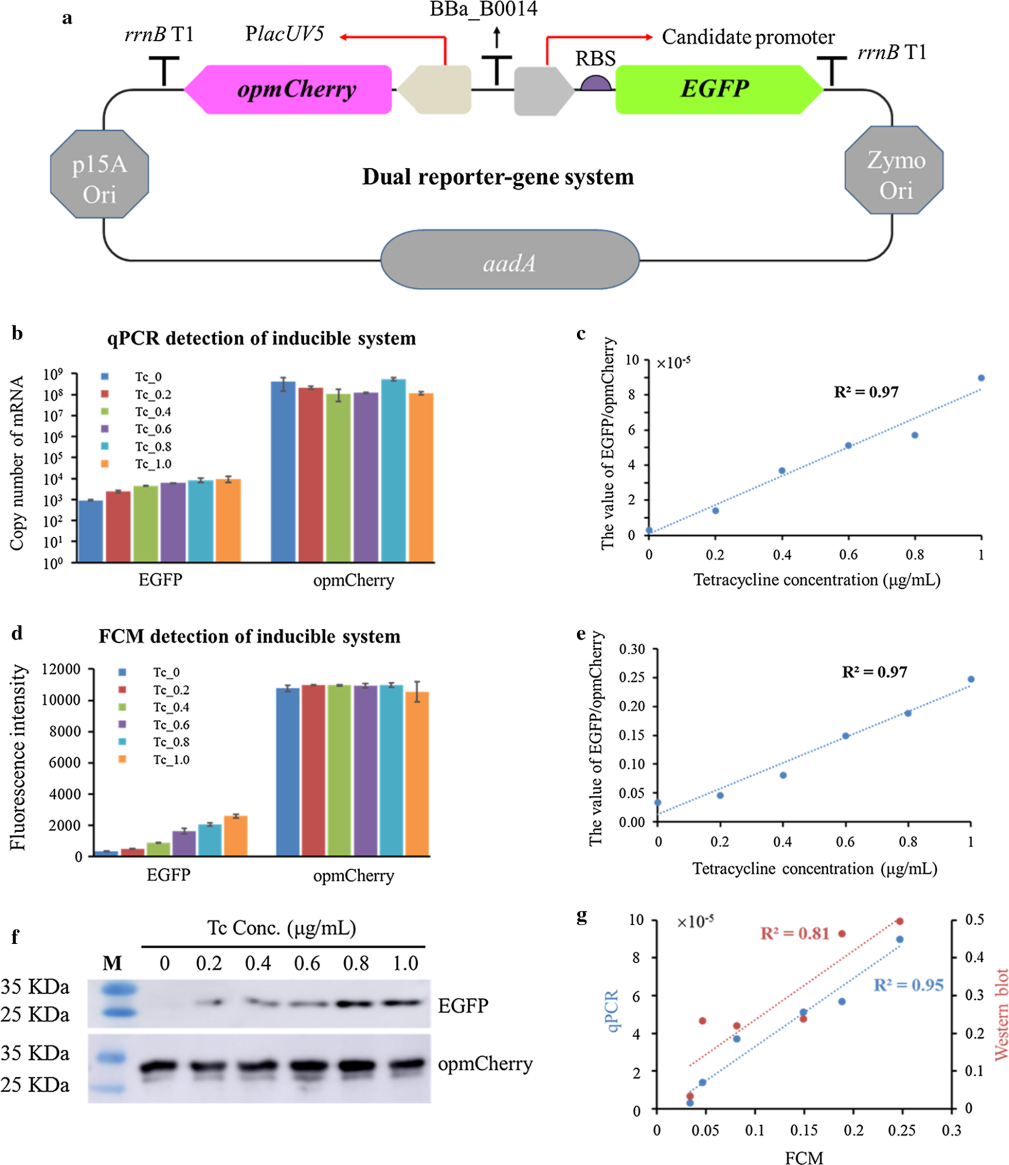
The schematic illustration of the dual reporter-gene system based on the pEZ15A shutter vector, which includes the reporter gene *opmCherry* driven by the constitutive promoter P_*lavUV5*_ and terminated by *rrnB* T1 terminator as the intrinsic control, another reporter gene *EGFP* under the control of the targeted genetic element and terminated by *rrnB* T1 terminator, and a terminator BBa_B0014 inserted between these two reporter-gene expression cassettes (**a**); evaluation of the dual reporter-gene system under P*tet* inducible system using different approaches of qPCR (**b**, **c**), flow cytometry (FCM, **d**, **e**) and Western blot (**f**), as well as the correlations of FCM results with qPCR and Western blot results, respectively (**g**). The concentrations of tetracycline are 0, 0.2, 0.4, 0.6, 0.8, or 1.0 μg/mL, respectively

Since opmCherry (excitation maximum 587 nm, emission maximum 610 nm) and EGFP (excitation maximum 488 nm, emission maximum 507 nm) are spectrally distinguishable with good photostability [59, 60] and relatively comparable faster mature rates (40 min and 25 min, respectively) [59, 61] than that of RFP (100 min) [62], they were then selected to construct the dual reporter-gene system with *opmCherry* driven by the constitutive promoter P_*lavUV5*_ as the intrinsic control and *EGFP* under the control of the targeted genetic element, such as the candidate promoter or RBS (Fig. 2a).

To confirm whether the dual reporter-gene system works, we first tested the system using an inducible promoter P*tet* to simulate the strengths of different promoters and determined the appropriate tetracycline inducer concentration. Our results indicated that the cellular growth decreased when tetracycline concentration was greater than 2 μg/mL, whereas the tetracycline concentration within 0–1 μg/mL did not affect cellular growth significantly (Additional file 2: Figure S1).

The correlation between the concentration of tetracycline inducer and the promoter strength was then investigated. The ratio of EGFP fluorescence intensity for tested promoter versus the opmCherry calibration fluorescence intensity was used to represent the promoter strength. qPCR, Western blot and flow cytometry analyses were carried out to evaluate the target gene expression at the transcriptional and translational levels. The results indicated that the expression of *opmCherry* is at a relatively constant level, whereas the expression of *EGFP* was enhanced correspondingly to the increase of the tetracycline inducer concentration within 0–1 μg/mL (Fig. 2b, d, f). In addition, the value of EGFP/opmCherry, a normalized ratio to eliminate the internal and external noises [55], has a linear correlation with the gradient tetracycline concentrations (Fig. 2c, e).

The correlations between the results of flow cytometry and other technologies, such as qPCR or Western blot, were also relatively high (Fig. 2g), which supports the possibility of utilizing the high-throughput quantitative approach of flow cytometry to quantify different candidate genetic elements.

### Identification of candidate promoters with different strengths using omics datasets

Systems biology datasets from 109 microarrays, eight RNA-seq, and four proteomic experiments of previous studies were used to identify potential promoters with different strengths. The strength of upstream promoters was presented as the normalized average value of downstream gene expression in normal conditions of each omics dataset. To select promoters with different strengths, we sorted the normalized value and defined strength of promoters arbitrarily based on their quantile values: in each omics dataset, those sorted in the top 90% were defined as strong promoters, those sorted in the last 10% were defined as weak promoters, and those sorted within 40–60% were defined as medium ones.

**Fig. 3:**
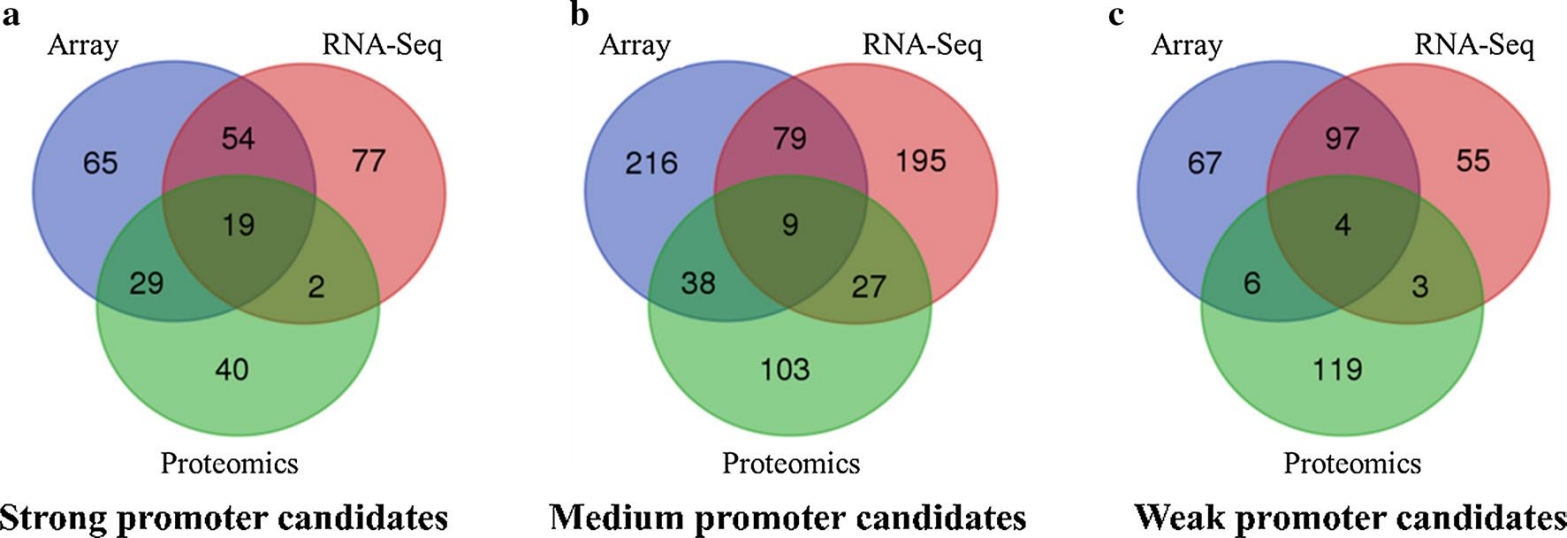
Identification of promoter candidates with different strengths of strong (**a**), medium (**b**), or weak (**c**) using the omics data of array, RNA-Seq, and proteomics. The sorted normalized values were used to defined the strength of promoters arbitrarily based on their quantile values: in each omics dataset, those sorted in the top 90% were defined as strong promoters, those sorted in the last 10% were defined as weak promoters, and those sorted within 40–60% were defined as medium ones. For a broad representation of weak promoters, six other weak promoters closing to the selection threshold were also included for characterization

Then, hierarchical cluster analysis cross different omics data was used to identify genes with consistent expression patterns (strong, medium, or weak), as shown in Venn diagram (Fig. 3). Six other weak promoters were also selected for a broad representation of weak promoters. Considering the existence of operon may affect the determination of the location for its upstream promoter, we used DOOR2 to predict the operons [63, 64]. A total of 38 candidate promoters with diverse strengths from strong to weak were then selected, which include 19 strong, nine medium, and ten weak ones (Table 1). In this study, the entire intergenic region of those genes was extracted as candidate promoters. These candidate promoters are listed in Table 1, and the information of promoter sequence and length are listed in supplementary materials (Additional file 1: Table S1).

**Table 1:** Candidate promoters with different strengths selected based on omics datasets

Gene ID	Gene name	Operon	Gene function	Array	RNA-Seq	Proteomics	EGFP/opmCherry (log phase)	EGFP/opmCherry (sta phase)
Candidate promoters with strong strength								
ZMO0177	*gap*		Glyceraldehyde-3-phosphate dehydrogenase, type I	15.06	11.2	9.23	0.38 ± 0.0202	0.47 ± 0.0191
ZMO1360	*pdc*		Thiamine pyrophosphate protein TPP binding domain-containing protein	14.52	11.78	8.59	0.24 ± 0.0220	0.22 ± 0.0086
ZMO0516	*Tuf*	ND	Elongation factor Tu	15.33	11.58	8.08	0.24 ± 0.0162	0.30 ± 0.0148
ZMO1608	*eno*		Phosphopyruvate hydratase	15.22	13.2	8.97	0.23 ± 0.0145	0.34 ± 0.0093
ZMO0997	*eda*	Yes	2-Dehydro-3-deoxyphosphogluconate aldolase/4-hydroxy-2-oxoglutarate aldolase	14.97	14.6	8.46	0.18 ± 0.0178	0.15 ± 0.0167
ZMO0367	*zwf*		Glucose-6-phosphate 1-dehydrogenase	14.92	11.19	7.38	0.16 ± 0.0057	0.14 ± 0.0124
ZMO1719	*frk*		ROK family protein	15.06	12.2	6.85	0.12 ± 0.0038	0.17 ± 0.0098
ZMO1609			Hypothetical protein	15.26	12.77	5.78	0.12 ± 0.0061	0.14 ± 0.0038
ZMO0689	*gfo*		Oxidoreductase domain-containing protein	14.49	11.68	6.38	0.10 ± 0.0075	0.13 ± 0.0130
ZMO1721	*gloA3*	ND	Glyoxalase/bleomycin resistance protein/dioxygenase	14.35	12.66	5.97	0.09 ± 0.0044	0.11 ± 0.0022
ZMO0514	*rpsG*	Yes	30S ribosomal protein S7	15.31	10.79	5.54	0.07 ± 0.0062	0.07 ± 0.0015
ZMO0515		Yes	Elongation factor G	15.07	11.14	5.83	0.07 ± 0.0062	0.07 ± 0.0015
ZMO1596	*adhB*		Iron-containing alcohol dehydrogenase	15.32	10.98	7.07	0.07 ± 0.0084	0.07 ± 0.0030
ZMO1141	*ilvC*	Yes	Ketol-acid reductoisomerase	15.41	12.39	6.76	0.05 ± 0.0012	0.04 ± 0.0006
ZMO0241	*atpD*	Yes	F0F1 ATP synthase subunit beta	15.09	11.73	7.77	0.05 ± 0.0030	0.04 ± 0.0029
ZMO0244			Histone family protein DNA-binding protein	14.79	12.98	6.1	0.04 ± 0.0013	0.04 ± 0.0007
Po1721							0.04 ± 0.0020	0.03 ± 0.0007
ZMO0493	*glnA*	Yes	Glutamine synthetase, type I	14.53	10.02	6.17	0.03 ± 0.0023	0.03 ± 0.0005
ZMO1779		Yes	Antibiotic biosynthesis monooxygenase	15.08	11.24	7.52	0.02 ± 0.0005	0.02 ± 0.0008
Candidate promoters with medium strength								
ZMO1351	*clcD1*	Yes	Carboxymethylenebutenolidase	12.93	6.81	3.14	0.14 ± 0.0064	0.16 ± 0.0036
ZMO0056	*glmS*		Glucosamine–fructose-6-phosphate aminotransferase	12.93	6.91	2.45	0.12 ± 0.0021	0.11 ± 0.0024
ZMO0559			Hypothetical protein	12.68	6.75	3.1	0.11 ± 0.0064	0.08 ± 0.0039
ZMO1385			Toxic anion resistance family protein	12.83	6.93	2.55	0.06 ± 0.0012	0.05 ± 0.0018
ZMO0127		Yes	S1/P1 nuclease	12.84	7.11	3.13	0.05 ± 0.0015	0.05 ± 0.0014
ZMO1100		Yes	Nucleotidyl transferase	12.58	7.18	2.82	0.05 ± 0.0012	0.05 ± 0.0023
ZMO1392			Hypothetical protein	12.46	7.43	2.45	0.04 ± 0.0012	0.04 ± 0.0012
ZMO0326			7-cyano-7-deazaguanine reductase	12.65	7.41	2.7	0.03 ± 0.0018	0.04 ± 0.0012
ZMO0570	*prmA*		Ribosomal L11 methyltransferase	12.38	7.32	2.45	0.03 ± 0.0030	0.04 ± 0.0013
Candidate promoters with weak strength								
ZMO1231	*recJ*		Single-stranded-DNA-specific exonuclease RecJ	11.03	5.7	0.07	0.08 ± 0.0023	0.07 ± 0.0029
ZMO1980	*gidB*	Yes	Methyltransferase GidB	10.59	5.27	0.07	0.05 ± 0.0025	0.05 ± 0.0029
ZMO1484			UvrD/REP helicase	10.93	5.4	0.07	0.05 ± 0.0013	0.05 ± 0.0026
ZMO0145		Yes	Peptidase M28	11.37	4.98	0.07	0.04 ± 0.0018	0.04 ± 0.0006
ZMO0101			NAD-dependent epimerase/dehydratase	10.61	5.05	0.07	0.04 ± 0.0013	0.04 ± 0.0014
ZMO1194	*dprA*	Yes	DNA protecting protein DprA	10.7	4.77	0.07	0.04 ± 0.0011	0.03 ± 0.0006
ZMO1644			DEAD/DEAH box helicase domain-containing protein	10.61	4.98	0.07	0.03 ± 0.0013	0.03 ± 0.0007
ZMO1582			Uracil-DNA glycosylase superfamily protein	10.33	4.63	0.07	0.03 ± 0.0004	0.03 ± 0.0003
ZMO0005	*cysD*	Yes	Sulfate adenylyltransferase subunit 2	11.5	5.28	0.07	0.03 ± 0.0012	0.03 ± 0.0009
ZMO0300	*xseA*		Exodeoxyribonuclease VII large subunit	11.6	4.71	0.07	0.03 ± 0.0010	0.03 ± 0.0013

The operon information is predicted by DOOR2 prediction server. The values of array, RNA-Seq and proteomics of each gene are the average log_2_-transformed values under different growth conditions

*ND* non-determined, *Log phase* exponential phase, *Sta phase* stationary phase

### Characterization of candidate promoters quantitatively using flow cytometry

Selected candidate promoters were assembled into the upstream of EGFP reporter gene in the dual reporter-gene system using a modified Gibson assembly approach [65]. The relative strength of these promoters was quantified in exponential and stationary phase by flow cytometry (Table 1). Each sample was operated in triplicates with at least two independent experiments conducted by two researchers in the lab at different times.

The results showed that P*gap*, P*pdc*, and P*eno* have high EGFP/opmCherry ratios as determined by our dual reporter-gene system, which actually is consistent with our omics data-driven predictions and with previous studies that showed P*gap*, P*pdc*, P*eno* and P*adhB* are strong promoters in *Z. mobilis* [42, 43, 66]. The P*gap*, encoding the glyceraldehyde-3-phosphate dehydrogenase (ZMO0177) in glycolysis pathway, is the strongest promotor in *Z. mobilis* characterized in this study (Table 1). Other promoters driving genes in glycolysis pathway also have high EGFP/opmCherry ratios (Table 1).

**Fig. 4:**
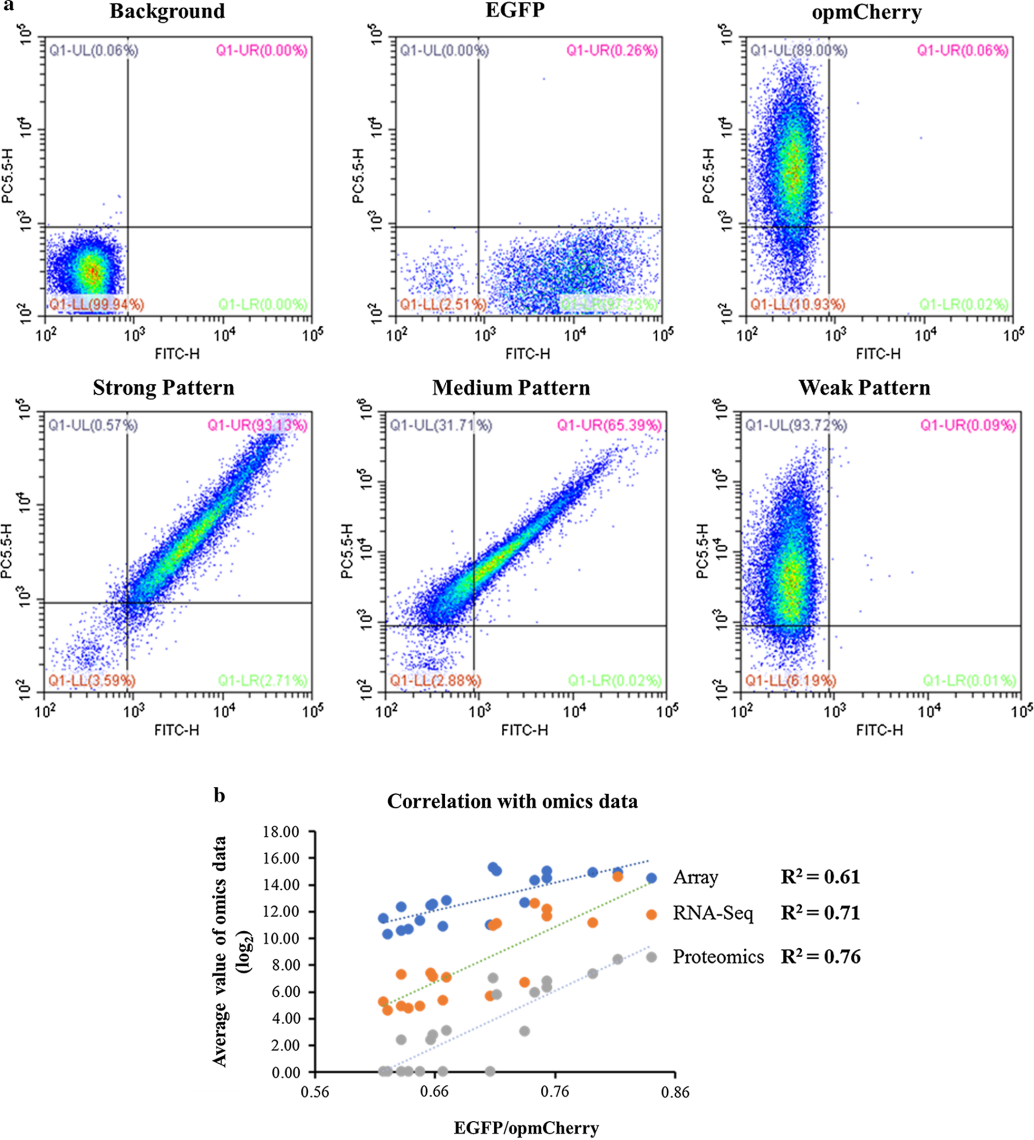
Flow cytometry (FCM) patterns of different promoters, in which the *X*- and *Y*-axis represents the fluorescent signal of EGFP and opmCherry, respectively. The background represents the fluorescence of the strain harboring an empty vector pEZ15Asp; EGFP and opmCherry represent the fluorescence of the strain harboring Plac*UV5* driving single reporter gene, *EGFP* and *opmCherry,* respectively. The strong, medium and weak pattern is the pattern of representative promoters of ZMO0177, ZMO1351, and ZMO0300, respectively (**a**); and the correlation of experimental data of EGFP/opmCherry ratios in log phase with log_2_-based values of omics data of array, RNA-Seq and proteomics, respectively (**b**)

Our results demonstrated that different flow cytometry profiles were identified and can be classified into strong, medium, and weak categories (Fig. 4a). This result suggested that the flow cytometry-based dual reporter-gene system can clearly distinguish different strengths of promoters based on their profiles. In addition, the correlations between omics data prediction and EGFP/opmCherry ratios are relatively high, with an *R*^2^ more than 0.7 for RNA-Seq and proteomics datasets (Fig. 4b).

### Quantification of predicted RBSs with different translation initiation rates

The dual reporter-gene system developed in this study was further explored to measure the RBSs with different translation initiation rates. The promoter used in this experiment was the inducible P*tet*; however, the RBS sequence was replaced by the synthesized RBS sequences with different translation initiation rates predicted by RBS calculator V2.0 (https://salislab.net/software/), which is based on the 16S rRNA sequence of *Z. mobilis*. From weak to strong translation initiation rates, four synthetic RBSs (Table 2) were selected for quantification in *Z. mobilis* using the dual reporter-gene system (Fig. 2).

**Table 2:** The nucleotide sequences of synthetic RBS with different translation initiation rates based on the prediction using RBS calculator (https://salislab.net/software/forward)

Name	Strength	Predicted RBS sequence (5′–3′)
ZM4-Ptet-GFP-10	10	CCATAATCTAGAGAAAGTAAGCAC
ZM4-Ptet-GFP-1000	1000	AGGCTAAGAACTAACGGAGAGGTAAAT
ZM4-Ptet-GFP-10000	10,000	ATCACAGGGTCTAGAAGGAGGTCGAA
ZM4-Ptet-GFP-Max	15,000	GAGCGAGAAGGAGGTAAAGT

**Fig. 5:**
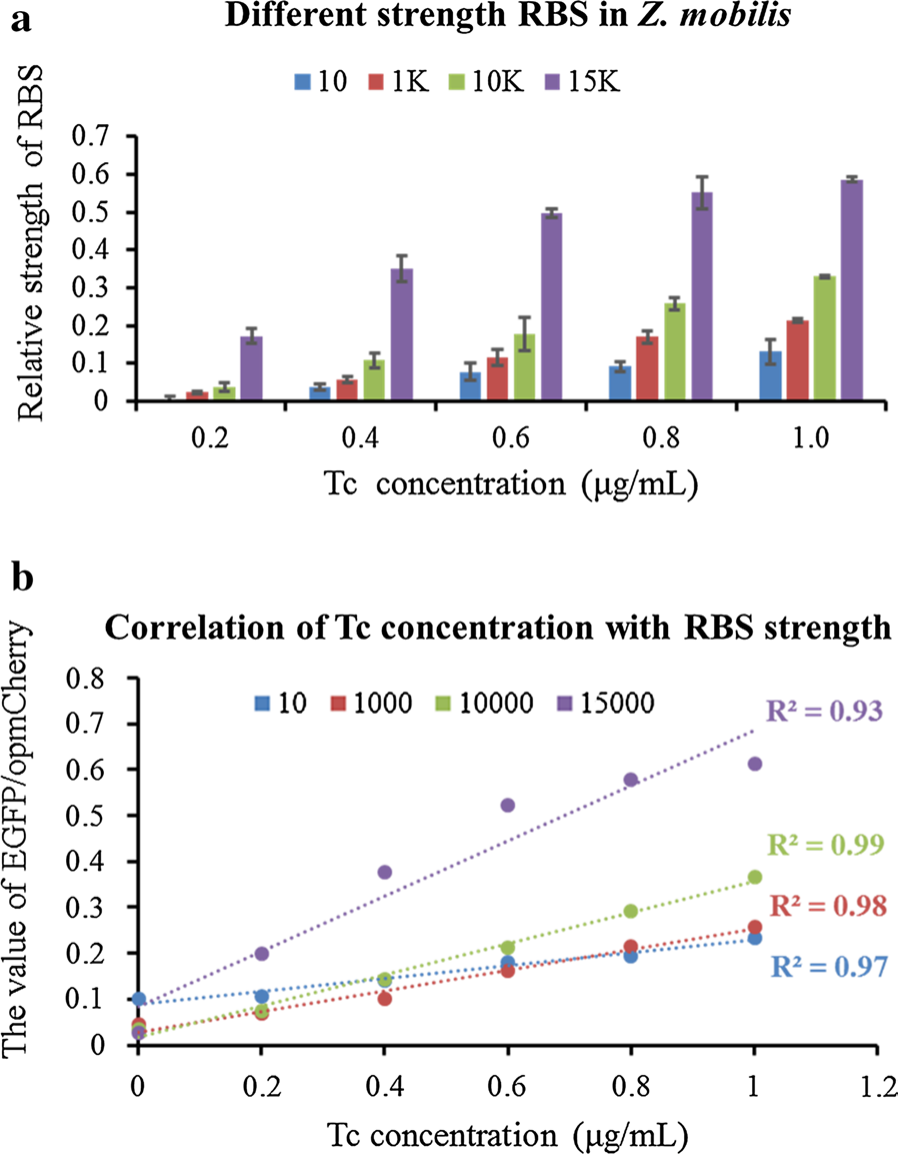
The quantification of synthetic RBS strengths by dual reporter-gene system under the induction of different concentrations of tetracycline (Tc). The relative strength of RBS is a calibrated strength that the value of EGFP/opmCherry with Tc induced minus that without Tc induction (**a**), and the correlation between tetracycline concentrations and RBS strengths of EGFP/opmCherry ratios for each predicted RBS with different strengths of 10, 1000, 10,000 and 15,000, respectively (**b**). The concentrations of tetracycline (Tc) used were 0, 0.2, 0.4, 0.6, 0.8, or 1.0 μg/mL, respectively

The results showed that within a fixed strength of translation initiation rate, the relative strength of RBS was enhanced with the increase of tetracycline inducer concentration gradient (Fig. 5a, Additional file 2: Figure S2). In addition, the predicted RBS strengths and tetracycline concentrations also had relatively high correlations with an *R*^2^ value of more than 0.93 (Fig. 5b). These results demonstrated that the dual reporter-gene system performed well in characterizing the strength of RBS, which suggests that the dual reporter-gene system can be used to characterize the non-coding sequence elements of RBSs.

## Discussions

In this study, a flow cytometry-based dual reporter-gene system was developed (Fig. 1), which was confirmed with an inducible promoter Ptet using different approaches of qPCR, flow cytometry, and Western blot (Fig. 2), and then applied to quantify biological parts such as promoters and RBS predicted by bioinformatics approaches with good correlations (Figs. 3, 4, 5). The high correlation between different concentrations of the tetracycline inducer and EGFP/opmCherry ratios suggested the capability of this dual reporter-gene system to characterize the strengths of different genetic elements, and the relatively high correlations between predicted candidates and experimental results for promoters and RBSs also indicate that it is practicable to predict the strengths of genetic elements based on omics datasets and bioinformatics tools, and therefore can be used to guide the selection of promoters with different strengths at the transcriptional level in other microbial systems.

Additionally, our result indicated that the codon-optimized EGFP actually had a decreased fluorescence, while the codon-optimized mCherry had enhanced fluorescence intensity (Fig. 1b). This system can, therefore, be applied to select and test reporter genes with desired fluorescence through protein engineering approaches such as codon optimization, rational design, and directed evolution.

Furthermore, this approach is straightforward with high reproducibility, and the fluorescence intensity of both EGFP and opmCherry as well as the corresponding correlation between results from different researchers in our group at different operation times is high with an *R*^2^ value of 0.92 (Additional file 2: Figure S3). However, future work is still needed to further develop this dual reporter-gene system for broad applications such as the inclusion of short-life reporter genes and advanced imaging techniques to monitor the dynamic expression changes of diverse genetic elements including promoters and RBSs investigated in this work as well as other genetic elements to be explored such as UTRs, terminators, and sRNA.

## Conclusion

In this study, we conducted the systems biology data mining and bioinformatics analyses to predict the strengths of genetic elements, such as promoters and RBSs. We also developed a flow cytometry-based dual reporter-gene platform for *Z. mobilis* to measure the strengths of selected promoters and synthesized RBSs. We found a relatively high correlation between prediction and experimental results for the strengths of promoters and RBSs. The feasibility of identifying and characterizing predicted genetic elements, such as promoters and RBSs, demonstrated in this study suggests that other genetic elements from *Z. mobilis*, such as 5′UTRs and terminators, could also be quantitatively characterized by similar approaches. Furthermore, the high correlation between prediction and experimental results indicates that a similar strategy could be applied to other microorganisms leading to the identification of genetic elements using systems biology data and bioinformatics tools. Moreover, the seven native and synthesized fluorescent proteins and genetic elements including 38 promoters and four RBSs characterized in this study can be used for future metabolic engineering and synthetic biology practices in *Z. mobilis*.

## Methods

### Strains, vectors, and media

*Escherichia coli* XL10-Gold from Invitrogen (USA), was cultured in Lysogeny broth (LB, 10 g/L NaCl, 10 g/L tryptone, 5 g/L yeast extract). Medium was prepared according to the description in the Manual of Molecular Cloning [67]. Wild type *Z. mobilis* ZM4 was revived from frozen glycerol stocks in rich media with 5% glucose (RMG5: 50 g/L glucose, 10 g/L yeast extract, and 2 g/L KH_2_PO_4_) at 30 °C for 6–8 h without shaking. Shuttle vector pEZ15Asp includes origins of replication from both *E. coli* and *Z. mobilis* [44]. Strains and plasmids used in this study are listed in Table 3.

**Table 3:** Strains and plasmids used in this study

Strains and plasmids	Description	Source
Strains		
*E. coli* XL10-Gold	Ultracompetent cells (tetracycline and chloramphenicol resistant)	Invitrogen
*Z. mobilis* ZM4	*Z. mobilis* wild-type strain	Lab stock
Plasmids		
pEZ15Asp	*P15A_ori*, *Zymo_Ori*, *P*_*tet*_, *Spe*^*R*^	[44]
pEZ15A-PlacUV5-EGFP	*P15A_ori*, *Zymo_Ori*, *P*_*lacUV5*_*::EGFP*, *Spe*^*R*^	This work
pEZ15A-PlacUV5-*op*mCherry	*P15A_ori*, *Zymo_Ori*, *P*_*lacUV5*_*::opmCherry*, *Spe*^*R*^	This work
pEZ-Dual	*P15A_ori*, *Zymo_Ori*, *P*_*tet*_, *Spe*^*R*^, *P*_*lacUV5*_*::opmCherry*, *P*_*tet*_*::EGFP*	This work

### Selection of candidate promoters

Public and in-house transcriptomic datasets from RNA-seq and microarray studies, as well as proteomic datasets were retrieved, collected, and hierarchically clustered separately using the JMP Genomics software 6.0 (SAS Inc., NC) with the conventional processing flows using default parameters [15, 16, 21, 23]. The common promoters showing strong, medium, or weak expression levels or abundance based on gene expression intensity or protein peptide hits abundance of downstream genes or proteins were selected using the Venn diagram generator at Venny 2.0.2 program (http://bioinfogp.cnb.csic.es/tools/venny/).

In this study, promoters were loosely defined as the region between the gene of interest and the preceding open reading frame (ORF). If genes were found to be transcribed as part of an operon, the operon promoter was selected. Genome-wide operon prediction in *Z. mobilis* is available at DOOR2 website server (http://csbl.bmb.uga.edu/DOOR/).

### Plasmid construction of candidate genetic elements

The vector pEZ15Asp containing an origin of replication with promoters for *E. coli* as well as *Z. mobilis* [44] was modified to construct the candidate genetic elements into the dual reporter-gene system. The reporter gene *opmCherry* driven by a constitutive promoter *LacUV5* was assembled to the suffix sequences of *Z. mobilis* replication promoter [68]. The reporter gene *EGFP* driven by tetracycline inducible promoter P*tet* was assembled to the prefix sequences of *E. coli* replication promoter.

For construction of candidate promoters into the dual reporter-gene system, the P*tet* was replaced by candidate promoters individually. All candidate promoters were PCR amplified from *Z. mobilis* genomic DNA using primer sets listed in Additional file 1: Table S2. Each primer contains about 15–20 nucleotides overlapping region of vector without P*tet* promoter. Assembly was operated as described below. Recombinant vectors were selected by colony PCR with primer pairs of Pdual-F and Pdual-R. The schematic diagram of dual-reporter system is shown in Fig. 2a. To construct RBSs into the dual reporter-gene system, the candidate RBSs replace the original RBS in the P*tet* dual reporter-gene system by same operation as described above.

For plasmid construction, the protocol used was based on Gibson et al. [65]. Briefly, primers were designed to contain 15–20 nucleotides overlapping regions with adjacent DNA fragments. PCR products amplified by primer pairs were separated by gel electrophoresis, followed by gel purification, and subsequently quantified using NanoDrop 2000 (Thermo Fisher Scientific, USA). Fragments and vector were allocated in a molar ratio of 3:1, 0.5 U T5 exonuclease (NEB, USA), 0.5 μL buffer 4 (NEB, USA), and the final volume was set to 5 μL with ddH_2_O.

All regents were mixed and reacted on the ice for 5 min; *E. coli* chemical competent cells were subsequently added. After incubation on the ice for 30 min, the mixture above was heat-shocked for 45 s at 42 °C, and then held on the ice for 2 min. Subsequently, 100 μL NZY was added into the mixture and incubated at least 1 h at 37 °C with shaking (250 rpm).

Cells were plated on LB agar plates containing spectinomycin, and recombinants were selected by colony PCR and confirmed by Sanger sequencing (Tsingke, China). The correct recombinant plasmids were transformed into *Z. mobilis* ZM4 competent cells, which were prepared as described previously [44], via electroporation (0.1-cm electrode gap, 1600 V, 200 Ω, 25 μF) using a Gene Pulser^®^ (Bio-Rad, USA). Colonies with correct PCR product sizes were selected as candidate strains.

### Flow cytometry analysis

The protocol used for flow cytometry analysis of the promoter strength in terms of fluorescence intensity was modified slightly from a previous study [69]. Briefly, cells were washed with phosphate-buffered saline (PBS) twice and then resuspended into PBS to a concentration of 10^7^ cells/mL. Cells were analyzed by flow cytometry using Beckman CytoFLEX FCM (Beckman Coulter, USA) with the PBS as the sheath fluid. The fluorescence of EGFP was excited with the 488 nm and detected with FITC; opmCherry was excited with the 561 nm and detected with PC5.5 [59, 70, 71].

As recommended by the manufacturer, compensation was applied to ensure that the EGFP has minimal affection on the detection of opmCherry. To avoid rare events which could affect the population distribution, at least 20,000 events of each sample were analyzed. Data were processed via FlowJo software (FlowJo, LLC, USA) based on the user manual with the recommended parameters. The mean fluorescence intensity of triplicates was calculated; then the ratio of average EGFP/average opmCherry was used to quantify the strength of each promoter. In addition, the standard deviation (STDEV) was set as the error bar.

### Quantitative real-time PCR analysis

The transcription levels of EGFP and opmCherry in the dual reporter-gene system were estimated at the same thermal cycling conditions. The cell extract samples were taken at exponential phase and used for quantitative real-time PCR (qPCR) analysis. Total RNA from *Z. mobilis* ZM4 strains harboring the inducible dual reporter-gene system was extracted using TRIzol reagent (Invitrogen, USA), and RNA quality was examined by NanoDrop 8000 (Thermo-Fisher, USA). The residual DNA removal and the reverse transcription was performed using the iScript™ gDNA Clear cDNA Synthesis Kits according to the manufacturer’s instructions (Bio-Rad, USA).

The qPCR reactions were carried out using iTaq™ Universal SYBR^®^ Green Supermix (Bio-Rad, USA) on a CFX96 Real-Time System (Bio-Rad, USA) as described previously [13, 15, 21, 23]. Briefly, highly purified salt-free primer pairs for EGFP and opmCherry were synthesized (Genscript, China) with an similar annealing temperature of 60 °C (Additional file 1: Table S1). PCR product specificity was confirmed through melting curve analysis. The following run protocol was used: 95 °C 5 min for denaturation; (95 °C 15 s, 60 °C 10 s, and 72 °C 30 s) 40 times for amplification and quantification with a single florescence measurement. A melting curve program (60–95 °C with heating rate of 0.1 °C per second and a continuous fluorescence measurement) was used to confirm the specificity of the primer pairs. An absolute quantification based on an internal calibration curve was applied for qPCR data analysis.

### Western blot analysis

Log phase and stationary phase cells were harvested to conduct the flow cytometry analysis. Cells were lysed and protein was extracted using Protein Extraction Kit (Zomanbio, China). Total protein concentrations of total cellular lysates were measured by the Bradford method with 200 ng of total protein loaded for each sample. Sodium dodecyl sulphate polyacrylamide gel electrophoresis (SDS-PAGE) was performed with a 5% stacking and a 12% running gel, followed by stained with Coomassie Brilliant Blue R-250. Molecular weight was estimated using a pre-stained protein ladder (10–170 kDa, Thermo, Lithuania) [72].

For Western blot analysis, after the electrophoresis, gels were transferred to methanol-activated PVDF membranes using the Trans-Blot^®^ Semi-Dry Electrophoretic Transfer Cell (Bio-Rad, USA) and run for 20 min at 25 V. PVDF membranes was then blocked with 5% non-fat milk in phosphate-buffered saline with Tween 20 (PBST) for 1 h at room temperature, and subsequently EGFP or opmCherry were probed with primary antibody (1:5000, Proteintech, China), respectively. Peroxidase-conjugated goat anti-Mouse IgG (1:5000, Proteintech, China) was used as secondary antibodies. Color development was performed by West Dure Extended Duration Substrate Kit (AntGene, China). All images were visualized using AI600 Imaging System (GE, USA), and analyzed by ImageJ (National Institutes of Health, USA).

## Additional files

**Additional file 1: Table S1.** Nucleotide sequences of promoters with different strengths and their gene name and function. **Table S2.** Primers used in this study. The lowercases in each primer are the homologous arms designed to assembly with the vector. The primers prefixed with Q are used in qPCR.

**Additional file 2: Figure S1.** Determination of the concentration of tetracycline based on the effect for cellular growth with a broad range of 0–50 μg/mL (A), and a narrow range of 0–1.0 μg/mL (B). **Figure S2.** Flow cytometry results of RBSs with different strengths in *Z. mobilis*. The concentrations of tetracycline are 0, 0.2, 0.4, 0.6, 0.8, or 1.0 μg/mL, respectively. The Ptet-RBS-ori represents the original Ptet-RBS sequence, and the Ptet-RBS-10, -1K, -10K and –Max represent the RBS sequences with different translation initiation rates of 10, 1000, 10000, and 15000 based on bioinformatics server RBS calculator. **Figure S3.** The fluorescence intensity of EGFP (A), and opmCherry (B), and the corresponding correlation between two repeat experiments by two individual researchers (C).

## Abbreviations

AFM: atomic force microscope
CFP: cyan fluorescent protein
EGFP: enhanced green fluorescent protein
EMSA: electrophoresis mobility shift assay
FCM: flow cytometry
NGS: next-generation sequencing
opCFP: codon-optimized CFP
opEGFP: codon-optimized EGFP
opmCherry: codon-optimized mCherry
PBS: phosphate-buffered saline
PBST: phosphate-buffered saline with Tween 20
qPCR: quantitative real-time PCR
RBS: ribosomal binding sites
RFP: red fluorescent protein
SDS-PAGE: sodium dodecyl sulphate polyacrylamide gel electrophoresis
UTR: untranslated region
