# Supplementary figures and images for: Prediction and characterization of promoters and ribosomal binding sites of Zymomonas mobilis in system biology era
Source: Biotechnol Biofuels. 2019 Mar 14;12:52. doi: 10.1186/s13068-019-1399-6 (PMC6417218; doi:10.1186/s13068-019-1399-6)

## Slide 1
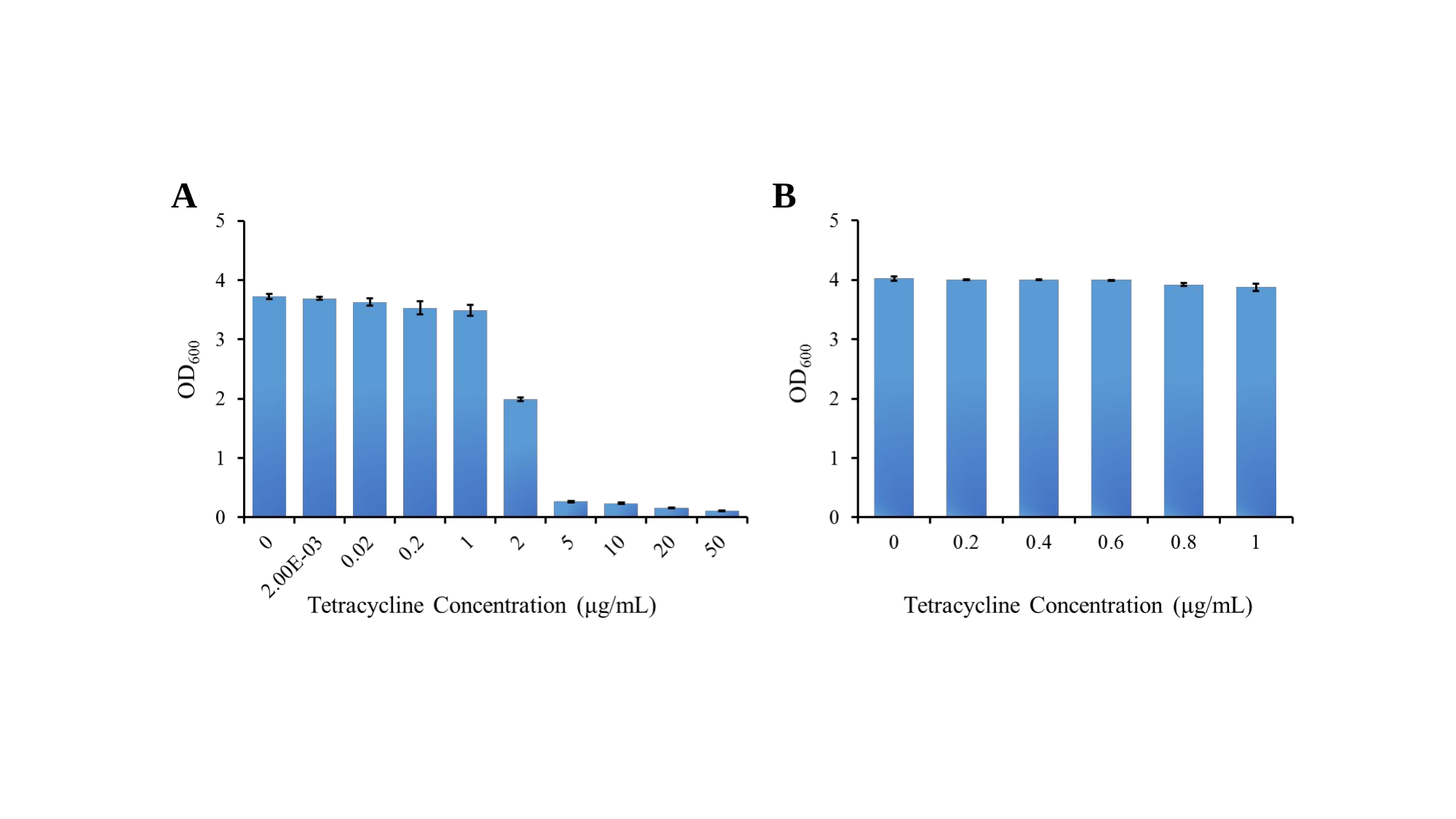

A
B

## Slide 2
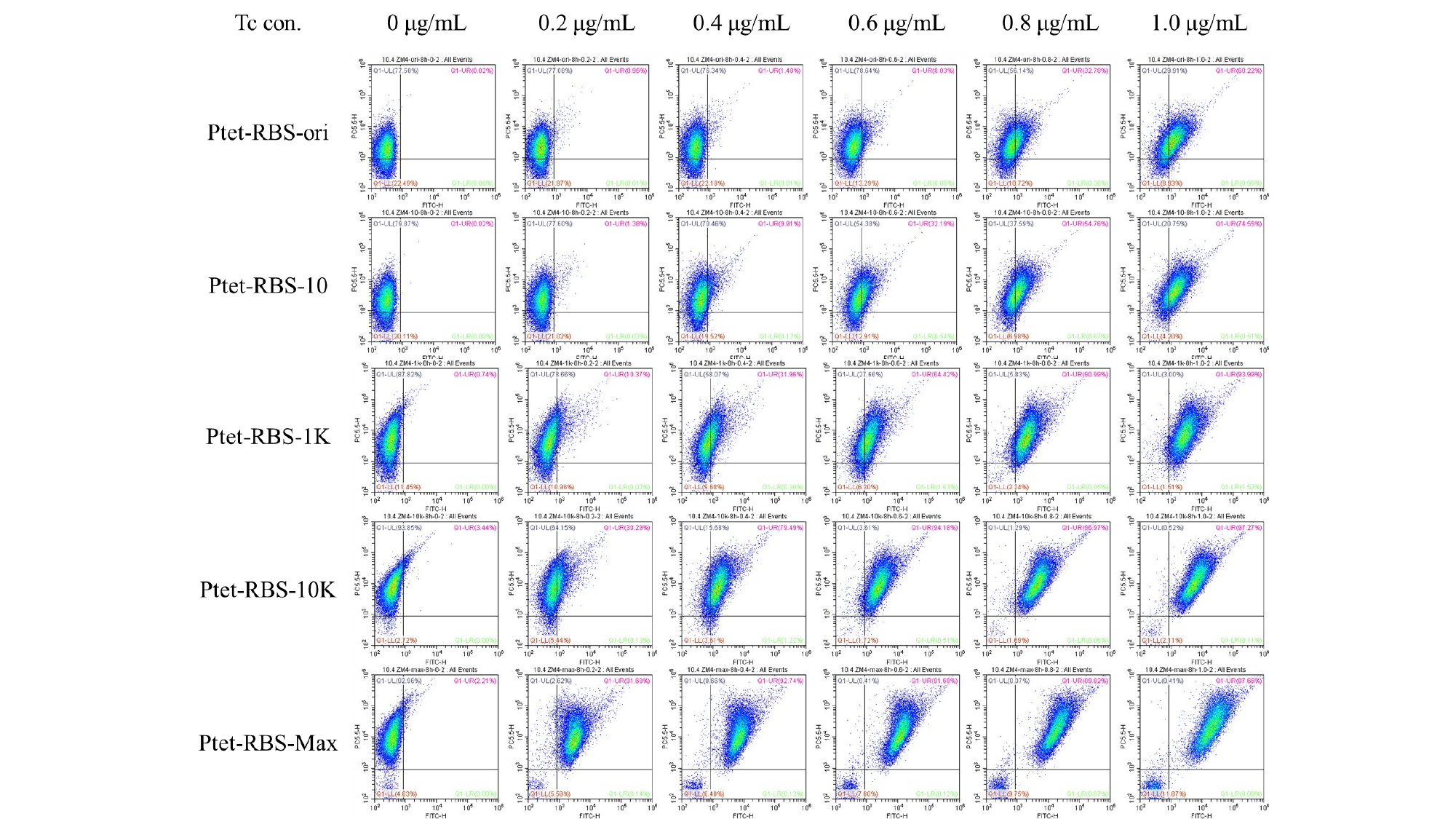

## Slide 3
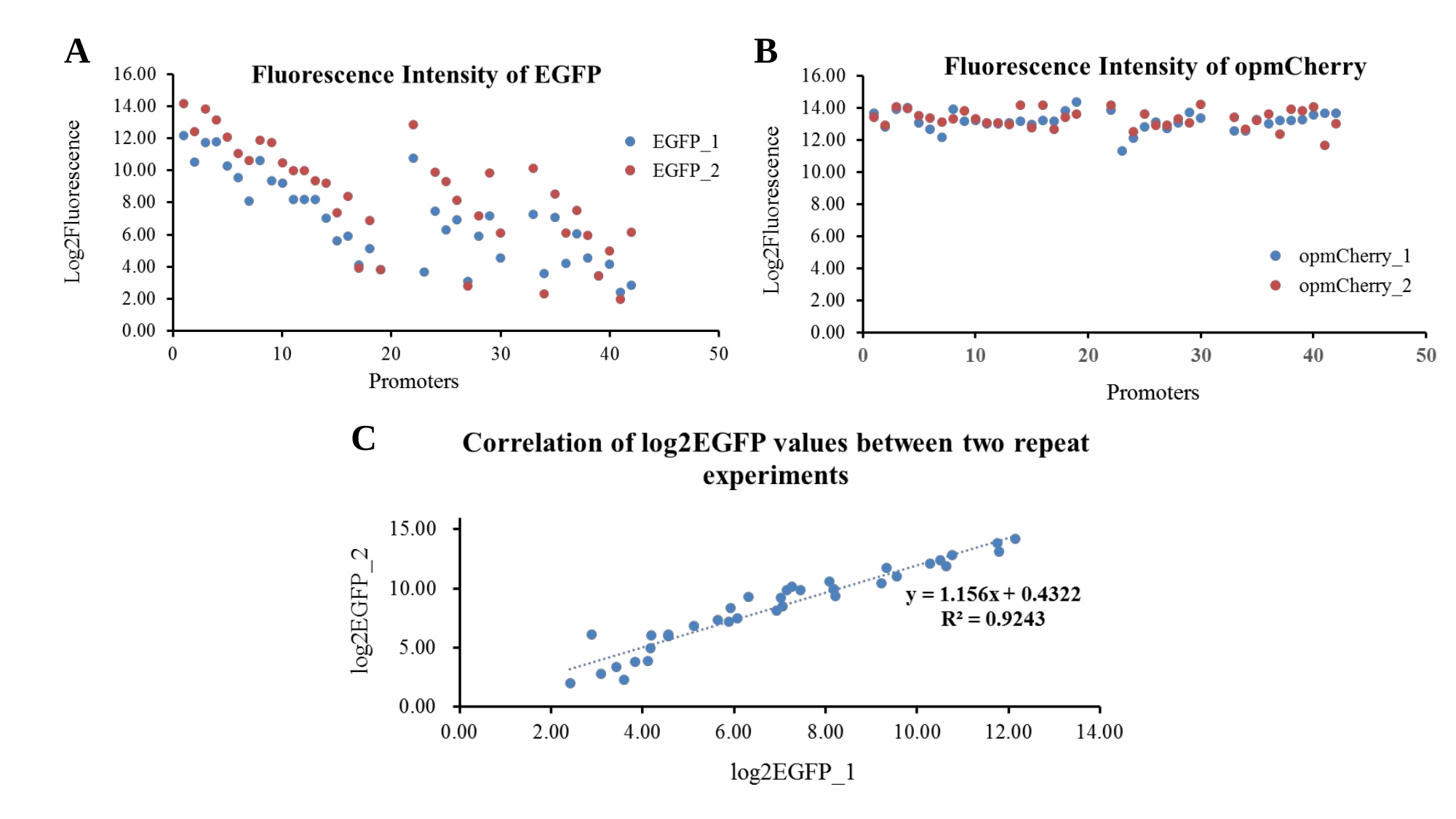

A
B
C

Supplement: Supplementary file 2 — Additional file 2: Figure S1. Determination of the concentration of tetracycline based on the effect for cellular growth with a broad range of 0–50 μg/mL (A), and a narrow range of 0–1.0 μg/mL (B). Figure S2. Flow cytometry results of RBSs with different strengths in Z. mobilis. The concentrations of tetracycline are 0, 0.2, 0.4, 0.6, 0.8, or 1.0 μg/mL, respectively. The Ptet-RBS-ori represents the original Ptet-RBS sequence, and the Ptet-RBS-10, -1K, -10K and –Max represent the RBS sequences with different translation initiation rates of 10, 1000, 10000, and 15000 based on bioinformatics server RBS calculator. Figure S3. The fluorescence intensity of EGFP (A), and opmCherry (B), and the corresponding correlation between two repeat experiments by two individual researchers (C). [file 13068_2019_1399_MOESM2_ESM.pptx]
